# Correction: Acridinedione as selective flouride ion chemosensor: a detailed spectroscopic and quantum mechanical investigation

**DOI:** 10.1039/d0ra90008g

**Published:** 2020-01-23

**Authors:** Nafees Iqbal, Syed Abid Ali, Iqra Munir, Saima Khan, Khurshid Ayub, Mariya al-Rashida, Muhammad Islam, Zahid Shafiq, Ralf Ludwig, Abdul Hameed

**Affiliations:** H. E. J. Research Institute of Chemistry, International Center for Chemical and Biological Sciences, University of Karachi Karachi-75270 Pakistan abdul_hameed8@hotmail.com +92-21-3481901 +92-21-99261701-2; Department of Chemistry, COMSATS Institute of Information Technology Abbottabad KPK Pakistan 22060; Department of Chemistry, Forman Christian College, A Chartered University Ferozepur Road Lahore Pakistan; Institute of Chemical Sciences, Bahauddin Zakariya University Multan Pakistan; Leibniz-Institut für Katalyse e. V. an der Universität Rostock Albert-Einstein-Str. 29a 18059 Rostock Germany; Department of Physical Chemistry, University of Rostock Dr.-Lorenz-Weg 1 18059 Rostock Germany; Department of Chemistry, Quaid-i-Azam University Islamabad 45320 Pakistan

## Abstract

Correction for ‘Acridinedione as selective flouride ion chemosensor: a detailed spectroscopic and quantum mechanical investigation’ by Nafees Iqbal *et al.*, *RSC Adv.*, 2018, **8**, 1993–2003.

The authors regret that the interpretation of the fluorescence spectra of compound 7i published in the original article was incorrect. In the original article, it was reported that upon excitation at 380 nm, the fluorescence spectrum of compound 7i showed two emission bands at 450 nm and 770 nm (Fig. 5b of the original article). The signal at 770 nm (previously reported as an emission band), is instead a second order diffraction (an artefact of diffraction grating/spectrofluorometer monochromator), as revealed from the literature.^1,2^ The authors thank a reader for highlighting this mistake.

The Royal Society of Chemistry apologises for these errors and any consequent inconvenience to authors and readers.

## Supplementary Material
